# Supplementary material for: Multispecies coalescent and its applications to infer species phylogenies and cross-species gene flow
Source: Natl Sci Rev. 2021 Jul 15;8(12):nwab127. doi: 10.1093/nsr/nwab127 (PMC8692950; doi:10.1093/nsr/nwab127)
Supplement: nwab127_Supplemental_File [file nwab127_supplemental_file.pdf]

Multispecies Coalescent and its Applications to Infer Species Phylogenies and Cross-Species Gene Flow

Xiyun Jiao,[1,2] Tomáš Flouri[1] and Ziheng Yang[1,*]

[1]Department of Genetics, Evolution and Environment, University College London, UK

[2]Department of Statistics and Data Science, Southern University of Science and Technology, China

***Corresponding author:** E-mail: z.yang@ucl.ac.uk.

Associate Editor: xxx xxx

Abstract

Multispecies coalescent (MSC) is the extension of the single-population coalescent model to multiple species. It integrates the phylogenetic process of species divergences and the population genetic process of coalescent, and provides a powerful framework for a number of inference problems using genomic sequence data from multiple species, including estimation of species divergence times and population sizes, estimation of species trees accommodating discordant gene trees, inference of cross-species gene flow, and species delimitation. In this review, we introduce the major features of the MSC model, discuss full-likelihood and heuristic methods of species tree estimation, and summarize recent methodological advances in inference of cross-species gene flow. We discuss the statistical and computational challenges in the field and research directions where breakthroughs may be likely in the next few years.

Key words: anomaly zone, BPP, deep coalescence, gene flow, Markov chain Monte Carlo, multispecies coalescent, species tree

Introduction

Developed in the 1980s, the coalescent is a stochastic process that describes the genealogical history of a sample of DNA sequences taken from a population [1–3]. Whereas traditional population genetic models of drift and mutation describe changes in allele frequencies over generations in the *population*, the coalescent focuses on the *sample* and traces the genealogical history of lineage joining of the sampled sequences backwards in time. The coalescent model is in particular suited to inference using genetic sequence data [4–7].

The multispecies coalescent (MSC) is an extension of the single-population coalescent to the case of multiple species [8]. It integrates the process of species divergences and the within-population process of drift and mutation. Placing the coalescent in the context of a species phylogeny makes it possible to use the ever-increasing genomic sequence data from multiple species to address a number of important biological questions, and in the past two decades, the MSC has emerged as the natural framework for such inferences. These include estimation of population parameters (such as species divergence times, population sizes for extant species and extinct ancestors, and rates of cross-species gene flow), estimation of species phylogeny accommodating heterogeneous gene genealogies across the genome, and delineation of species boundaries (species delimitation) [9–12]. In molecular phylogenetics, incorporation of the MSC to accommodate the so-called gene-tree–species-tree conflicts has been heralded as a "paradigm shift" [13]. Stochastic fluctuation in genealogical history of sequences across the genome, when accommodated in the model, is not a 'conflict' or 'problem', but rather a source of information for important evolutionary parameters such as ancestral population sizes [14–16] and rates of cross-species gene flow [17, 18].

The past decade has seen exciting advancements in the implementation and extension of the MSC model for inference using genomic sequence data. The data we consider in this review are sequence alignments at hundreds or thousands of loci, with the different loci having independent coalescent histories while all sites in the sequence at the same locus share the same history. Ideal data for such analysis are short segments sampled from the genome that are far apart [16]. While we use the term gene or locus, the data should ideally be noncoding DNA, although exonic data have been successfully used in such analyses [19, 20]. We describe the major features of the MSC model (in particular, the probability distribution of gene trees and coalescent times), and discuss its applications in two major areas: the estimation of the species phylogeny and the inference of cross-species gene flow. We focus on full-likelihood methods (maximum likelihood or ML and Bayesian

© The Author 2021.

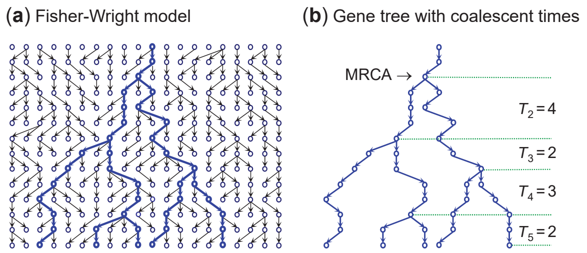

FIG. 1. The Fisher-Wright model for a diploid population of N individuals or $2N = 20$ sequences, with $n = 5$ sequences sampled at random from the present generation. The coalescent focuses on the genealogical relationships among the sampled sequences (in blue). Coalescent time T_i (during which there are i lineages in the sample) is in generations.

inference), as they have the best statistical properties, but include heuristic methods based on summaries of the data in our discussion. Several comprehensive reviews on heuristic methods have been published [9, 10, 21–23]. We review recent advances in using the MSC model to infer ancient gene flow, including models of continuous migration (the so-called isolation-with-migration or IM model) and the introgression/hybridization models. We end the paper with a discussion of the challenges and perspectives in the field. Our focus in this review is on MSC-based analyses of multilocus sequence data, and we do not consider population genetics methods that use summary statistics such as allele frequencies and single nucleotide polymorphisms (SNPs) to infer demographic processes including population structure and admixture [24, 25].

Multispecies Coalescent

Fisher-Wright model and the coalescent

The Fisher-Wright model [26, 27] in population genetics describes the biological process of reproduction and drift in an idealized population of constant size, with non-overlapping generations, random mating, and no population structure or selection (fig. 1a). Individuals of the next generation are generated by random sampling of gametes from the current population: the frequencies of alleles at a locus (say, A and a for two alleles) in the next generation are generated by binomial sampling given the allele frequencies in the current generation.

The *coalescent* model describes the same process of reproduction and drift, with the focus on the sample of sequences and with time running backwards (fig. 1b) [1]. When we trace the the genealogical history of the sample backwards in time, lineages join or coalesce when we reach their common ancestors. While the forward Fisher-Wright model and backward coalescent model are two characterizations of the same process, the coalescent approach of focusing on the sample offers major advantages for many inference problems using genetic sequence data. For example, coalescent simulation of the genealogy of the sample is often far more efficient than forward simulation tracking the whole population. The basic coalescent model has been extended to accommodate demographic changes, recombination, population subdivision, and selection [5, 7]. Here we focus on the basic coalescent and on the probability distribution of gene tree topologies and coalescent times generated by the process.

Consider first $n = 2$ sequences sampled from a diploid population of size N. With random mating assumed in the Fisher-Wright model, sequences pick parents at random when we trace the genealogical history of the sample to the previous generation. As there are $2N$ parental sequences to choose from, the probability that the two sequences pick the same parent (that is, they coalesce) in the previous generation is $\frac{1}{2N}$. In other words, coalescent occurs as a Poisson process at the rate of $\frac{1}{2N}$, faster in smaller populations, and the coalescent time (the waiting time until the two sequences find their common ancestor) has a geometric distribution with the mean of $2N$ generations. Thus two sequences sampled at random are on average separated by $2N \times 2$ generations or $\theta = 4N\mu$ mutations per site, where μ is the mutation rate per site per generation. Parameter θ, known as the population size parameter, is the average distance between two sequences sampled at random from the population. It is also known as heterozygosity and can vary hugely even between close species. Typical values include $\theta \approx 0.1\%$ for the humans [28] and 0.1-5% for *Heliconius* butterflies [29].

In analysis of sequence data, it is convenient to measure time by the mutational distance so that one time unit is the expected time to accumulate one mutation per site. With this time unit, the coalescent waiting time for two sequences (t_2) is approximately exponential with the mean $\frac{\theta}{2}$, and density

$$f(t_2) = \tfrac{2}{\theta}\, \mathrm{e}^{-\frac{2}{\theta}t_2}. \qquad (1)$$

If there are $n > 2$ sequences in the sample, there will be $\binom{n}{2} = \frac{n(n-1)}{2}$ pairs and each pair coalesce at the rate of $\frac{2}{\theta}$, with the total rate $\binom{n}{2} \cdot \frac{2}{\theta}$. The time until the next coalescent event has an exponential distribution with mean $\frac{\theta}{2}/\binom{n}{2} = \frac{\theta}{n(n-1)}$. When a coalescent occurs, each of the $\binom{n}{2}$ pairs has the same probability to join. The number of lineages is then reduced from n to $n-1$, and the process repeats, until the most recent common ancestor (MRCA) is reached (fig. 1b).

The $n-1$ successive coalescent events generate a genealogical tree (G) of the sequences in the sample. This is a rooted tree with the internal nodes ranked by age, and is called the *ranked tree* or *labelled history* [30] (fig. 1b). The number of possible labelled histories for a sample of size n is $H_n = \prod_{i=2}^{n} \binom{i}{2} = \frac{n!(n-1)!}{2^{n-1}}$, and each of them occurs with equal probability, $f(G) = 1/H_n$. Furthermore, the $n-1$ coalescent times $\boldsymbol{t} = \{t_n, t_{n-1}, \ldots, t_2\}$ are independent exponential variables, with means $\mathbb{E}(t_i) = \frac{\theta}{2}/\binom{i}{2}$. The joint probability density of the gene

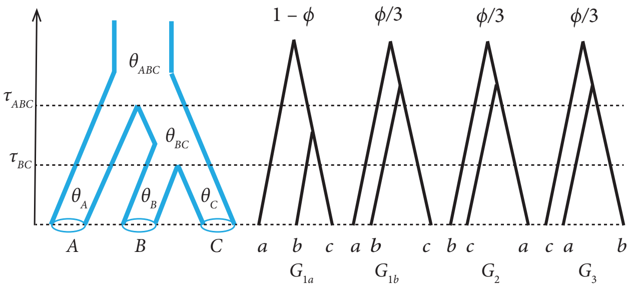

FIG. 2. A species tree for three species (A, B and C) showing parameters in the MSC model, and the four possible coalescent histories for a locus with one sequence from each species, with probabilities $\left(1-\phi, \frac{1}{3}\phi, \frac{1}{3}\phi, \frac{1}{3}\phi\right)$, where $\phi = \mathrm{e}^{-2(\tau_{ABC}-\tau_{BC})/\theta_{BC}}$ is the probability that sequences b and c do not coalesce in species BC. Note that the first two histories correspond to the same rooted gene tree G_1, and there are three gene trees: G_1, G_2, and G_3.

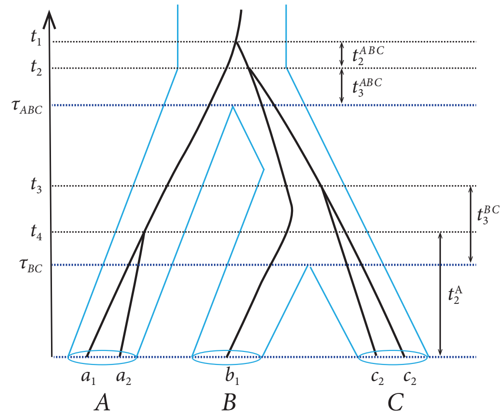

FIG. 3. A species tree for three species, $(A,(B,C))$, with a gene tree for five sequences at a locus to illustrate the MSC density of the gene tree with coalescent times.

tree and coalescent times is thus

$$\begin{aligned} f(G,\boldsymbol{t}) &= \frac{1}{\prod_{i=2}^{n}\binom{i}{2}} \times \prod_{i=2}^{n}\left[\binom{i}{2}\frac{2}{\theta}\exp\left\{-\binom{i}{2}\frac{2}{\theta}t_i\right\}\right] \\ &= \prod_{i=2}^{n}\frac{2}{\theta}\exp\left\{-\frac{i(i-1)}{\theta}t_i\right\}. \end{aligned} \tag{2}$$

Multispecies coalescent: basic features

The extension of the single-population coalescent to multiple species has been called the *interspecific coalescent* [31] or *censored coalescent* [8], and is now commonly known as the multispecies coalescent (MSC) [32]. Suppose there are s species, which are related through a species phylogeny. Instead of a single parameter θ, the model now involves two sets of parameters: $s-1$ species divergence times (τs) and $2s-1$ population size parameters (θs), with a total of $3s-2$ parameters (fig. 2). Both τs and θs are measured in the expected number of mutations per site.

Given the species tree, coalescent events occur independently in different populations, with the coalescent rate ($\frac{2}{\theta}$) given by the population size. When we trace the history of the sequences at a locus backwards in time and reach a speciation event, the coalescent process and rate are reset, because of the change in population size and because of sequences coming from the sibling species. For example, in figure 3, sequences c_1 and c_2 coalesce at the rate $\frac{2}{\theta_C}$ in species C. When they enter species BC at time τ_{BC}, the coalescent rate (for each pair) is reset to $\frac{2}{\theta_{BC}}$ and the number of lineages becomes 3. Furthermore, we assume that gene trees at different loci are independent. One important feature of the MSC model is that the divergence time between sequences from two species must be greater than the species divergence time: *sequences split before species* or equivalently *the gene tree fits inside the species tree*. This intrinsic constraint between the species tree and the gene trees is the source of computational challenges in Bayesian implementations of the MSC model.

There are two important probability distributions under the MSC model: the (marginal) probabilities of gene tree topologies [21, 33, 34] and the joint distribution of the gene tree topology and coalescent times [8]. The former is useful for two-step methods of species tree estimation, which use reconstructed gene tree topologies as data, while the latter is used in full-likelihood methods, which use information in gene-tree branch lengths (coalescent times) as well.

Probabilities of gene tree topologies

Under the MSC model, the gene tree topologies and coalescent times have a joint probability distribution given the species tree and parameters. For small species trees, it is easy to derive the marginal probability of gene tree topologies [2, 33, 35]. This line of work typically assumes one sequence sampled per species at every locus, so that there is no coalescent in modern species at the tips of the species tree. The case of three species is considered in [2]. Let the three species be A, B and C, with the phylogeny $S = (A,(B,C))$ (fig. 2). Let the divergence times be $\boldsymbol{\tau} = (\tau_{BC}, \tau_{ABC})$ and the population sizes be $\boldsymbol{\theta} = (\theta_{BC}, \theta_{ABC})$. Suppose three sequences are sampled from the three species (a, b and c). There are three possible gene tree topologies: $G_1 = (a,(b,c))$ matches the species tree, while $G_2 = (b,(c,a))$ and $G_3 = (c,(a,b))$ are the mismatching gene trees.

When we trace the genealogy of the three sequences, sequences b and c may coalesce in population BC as a Poisson event at the rate of $\frac{2}{\theta_{BC}}$ just as in the single-population coalescent. Note that the probability that a Poisson event of rate λ does not occur in a time interval t is $\mathrm{e}^{-\lambda t}$. Thus the probability that sequences b and c do not coalesce in population BC or over the time interval $\Delta\tau = \tau_{ABC} - \tau_{BC}$ is

$$\phi = \mathrm{e}^{-2\Delta\tau/\theta_{BC}} = \mathrm{e}^{-2(\tau_{ABC}-\tau_{BC})/\theta_{BC}}. \tag{3}$$

Here $\Delta\tau/\left(\frac{\theta_{BC}}{2}\right)$ is known as the *internal branch length in coalescent units*—one coalescent unit in population BC is $2N_{BC}$ generations or $\theta_{BC}/2$ mutations per site. If b and c coalesce in population BC, the gene tree must be G_1. Otherwise all three sequences enter species ABC and coalesce in random order so that the three gene trees

occur with equal probability. Thus the probabilities for the three gene trees (G_1, G_2, G_3) are

$$\begin{aligned}\mathbb{P}(G_1) &= (1-\phi) + \tfrac{1}{3}\phi = 1 - \tfrac{2}{3}\phi, \\ \mathbb{P}(G_2) &= \mathbb{P}(G_3) = \tfrac{1}{3}\phi.\end{aligned} \tag{4}$$

For certain species trees and parameter values, a mismatching gene tree may be more probable than the matching gene tree. The species tree is then said to be in the *anomaly zone* [33, 34]. The anomaly zone does not exist for species trees of three species — as $\mathbb{P}(G_1) > \mathbb{P}(G_2) = \mathbb{P}(G_3)$ in eq. 4, but can occur for asymmetrical species trees of four species, and for any species tree of five or more species [34].

Consider the asymmetrical species tree for four species $S = (A,(B,(C,D)))$ of figure 4, and suppose the three divergence times are very close, with $\tau_{ABCD} \approx \tau_{BCD} \approx \tau_{CD}$. Then all three coalescent events for the four sequences (a, b, c, and d) will most likely occur in the root population $ABCD$, so that the $18 = \binom{4}{2}\binom{3}{2}\binom{2}{2}$ labelled histories will have nearly equal probability $\frac{1}{18}$. There are 15 possible rooted gene trees, 12 asymmetrical and 3 symmetrical. Each symmetrical gene tree (e.g., G_2 in fig. 4) corresponds to two labelled histories (G_{2a} and G_{2b} in fig. 4), so that its probability is $\sim \frac{2}{18}$. Each of the 12 asymmetrical gene trees (e.g., G_1 in fig. 4) is compatible with only one labelled history, with probability $\sim \frac{1}{18}$. Thus $\mathbb{P}(G_2) \approx 2\mathbb{P}(G_1)$. When the divergence times (τs) are unequal but the internal branches are short enough, it is possible for the symmetrical mismatching gene tree G_2 to have a higher probability than the matching asymmetrical gene tree G_1, in which case the species tree is in the anomaly zone.

If the species tree is in the anomaly zone, the simple *majority-vote* approach of using the most commonly observed gene tree as the estimate of the species tree is statistically inconsistent: the more gene trees there are, the more certain that the species-tree estimate will be incorrect. Note that the existence of the anomaly zone is not an intrinsic difficulty for species tree estimation; it instead highlights the importance of adopting a proper statistical inference framework. Full likelihood methods are consistent for all species trees both in and outside the anomaly zone, as they accommodate the probability distribution of the gene trees under the MSC appropriately. The discussion of the anomaly zone typically assumes true gene trees and ignores phylogenetic reconstruction errors in estimated gene trees. There have been only a handful of empirical examples of the anomaly zone, in African *Anopheles* mosquitoes [20], skinks [36], flightless birds [37], and gibbons [19].

The probabilities of gene tree topologies can be used to calculate the likelihood function for estimating the species tree using (reconstructed) gene trees as input data, as in the STELLS program [38]. However, popular heuristic methods such as MP-EST [39] and ASTRAL [40] do not use this theory and are instead based on species triplets or quartets. Furthermore, calculation of the probabilities of gene tree topologies, which involves summing over all coalescent histories that are compatible with each gene tree, becomes expensive when the number of species increases [21].

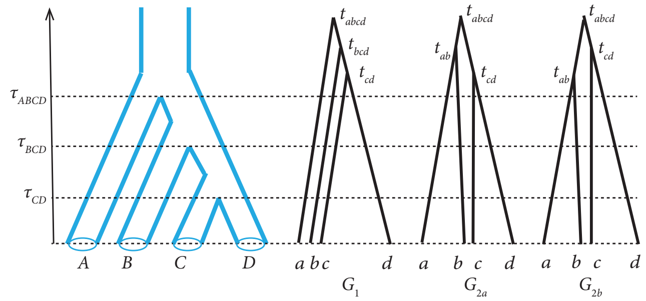

FIG. 4. Asymmetrical species tree for four species A, B, C and D, and three labelled histories (G_1, G_{2a}, G_{2b}) for a locus with one sequence from each species. G_1 matches the species tree, while G_{2a} and G_{2b} are distinct labelled histories sharing the same topology $((a,b),(c,d))$, which is different from the species tree.

Joint probability distribution of gene trees and coalescent times

While the marginal probability of the gene tree topology may be challenging to compute, it is straightforward to derive the joint distribution of gene tree topologies and coalescent times. The general form, for an arbitrary species tree and an arbitrary number of sequences, is given in [8].

The joint density of gene trees and coalescent times is a product over the populations on the species tree, and as a result we focus on the contribution from one population. A population is represented by a branch on the species tree (say XY) or by the daughter node of the branch (say X). Let τ_X and τ_Y be node ages or divergence times, and θ_X be the population size. Suppose m sequences enter the population at time τ_X and l sequences leave the population at time τ_Y, with $1 \le l \le m$. For example, in the gene tree of figure 3, $m = 3$ lineages enter population BC while $l = 2$ lineages leave it. Unlike the single-population coalescent, under the MSC, lineages entering a population do not necessarily find their common ancestor in that population, and the coalescent process may be 'censored' [8]. Note that if X is the root of the species tree, l must be 1.

The MSC density for the part of the gene tree residing in population XY is the product of three components. The first is the joint density of the $m-l$ independent exponential coalescent waiting times $\{t_m^X, t_{m-1}^X, \ldots, t_{l+1}^X\}$. The second component is for the gene tree topology in XY, and is a product of $m-l$ probabilities, each being the probability, $1/\binom{i}{2}$, of choosing two out of i lineages to join, for $i = m, m-1, \cdots, l+1$. These two components are the same as in the single-population coalescent. The third component is the probability that no coalescent events occur in the last time interval before reaching τ_Y. Multiplying the three components, we obtain the MSC

density of the gene tree in XY as

$$\left(\frac{2}{\theta_X}\right)^{m-l} \exp\Big\{-\sum_{i=l+1}^{m} \frac{i(i-1)}{\theta_X} t_i^X - \frac{l(l-1)}{\theta_X}\Big(\tau_Y - \tau_X - \sum_{i=l+1}^{m} t_i^X\Big)\Big\}. \tag{5}$$

For example, the contribution of species BC to the MSC density of the gene tree in figure 3 is

$$\frac{2}{\theta_{BC}} \exp\big\{-\frac{6}{\theta_{BC}} t_3^{BC} - \frac{2}{\theta_{BC}}\left(\tau_{ABC} - \tau_{BC} - t_3^{BC}\right)\big\}. \tag{6}$$

As coalescent processes in different populations operate independently, the MSC density for the whole gene tree at a locus is the product of the contributions across all populations. For the gene tree of figure 3, this is

$$\begin{aligned} f(G, \boldsymbol{t}|S, \Theta) = &\left[\frac{2}{\theta_A} \mathrm{e}^{-\frac{2}{\theta_A} t_2^A}\right] \times \left[\mathrm{e}^{-\frac{2}{\theta_C}\tau_{BC}}\right] \\ &\times \left[\frac{2}{\theta_{BC}} \mathrm{e}^{-\frac{6}{\theta_{BC}} t_3^{BC} - \frac{2}{\theta_{BC}}\left(\tau_{ABC} - \tau_{BC} - t_3^{BC}\right)}\right] \\ &\times \left[\frac{2}{\theta_{ABC}} \cdot \frac{2}{\theta_{ABC}} \mathrm{e}^{-\frac{6}{\theta_{ABC}} t_3^{ABC} - \frac{2}{\theta_{ABC}} t_2^{ABC}}\right]. \end{aligned} \tag{7}$$

The four pairs of brackets correspond to species A, C, BC and ABC, respectively. Coalescent is not possible in species B as only one sequence is sampled from that species.

With multiple loci in the data, the joint MSC density of the gene trees is a product across all loci, because the genealogical histories at different loci are assumed to be independent. The formulation allows the loci to have different sampling configurations. For example, the number of sequences from each species may vary among loci and some species may be missing at some loci.

Species Tree Inference under the MSC

Species-tree – gene-tree conflicts

The gene tree representing the coalescent history of the sequences at a locus may not match the species tree. Such a discordance may occur because when we trace the history of the sample backwards in time, sequences from different species may not coalesce as soon as they reach the most recent common ancestor on the species tree but instead coalesce in more ancient ancestors (e.g., gene trees G_{1b}, G_2, G_3 in fig. 2). This *delayed coalescence* or *deep coalescence* is also known as *incomplete lineage sorting* (ILS). While several biological processes, including gene duplication followed by gene loss or horizontal gene transfer [41, 42], can cause the gene tree to differ from the species tree as well, deep coalescence is more fundamental because coalescent is simply biological reproduction and drift and thus may affect every species. Deep coalescence is more common when multiple species arose through a rapid succession of speciation events resulting in very short internal branches on the species tree relative to the coalescent waiting time (note that ϕ in eq. 3 is greater for smaller $\Delta\tau$ and larger θ_{BC}). The existence of the anomaly zone is an extreme case of deep coalescence. Deep coalescence is related to how short the internal branches are, rather than how deep they are on the species tree, and may thus occur in both shallow and deep species trees [43].

Full likelihood methods

ML methods [44, 45] and Bayesian inference [46–49] use the joint distribution of gene trees and coalescent times [8] and operate on multilocus sequence data directly. Let the sequence data be $\boldsymbol{X} = \{X_j\}$, where X_j is the alignment of n_j sequences at the jth locus, for $j = 1, 2, \cdots, L$. Let S be the species tree and $\Theta = \{\boldsymbol{\tau}, \boldsymbol{\theta}, \eta\}$ be the vector of parameters, including species divergence times ($\boldsymbol{\tau}$), population sizes ($\boldsymbol{\theta}$), and parameters in the mutation model (η). The likelihood of the sequence data given the MSC model has the form

$$f(\boldsymbol{X}|S, \Theta) = \prod_{j=1}^{L} \sum_{G_j} \int_{\boldsymbol{t}_j} f(X_j|G_j, \boldsymbol{t}_j, \eta) f(G_j, \boldsymbol{t}_j|S, \Theta) \mathrm{d}\boldsymbol{t}_j, \tag{8}$$

where $f(X_j|G_j, \boldsymbol{t}_j, \eta)$ is the phylogenetic likelihood given the gene tree G_j and branch lengths $\boldsymbol{t}_j$ at locus j [50], while $f(G_j, \boldsymbol{t}_j|S, \Theta)$ is the MSC density of the gene tree described above [8]. As the genealogical histories at different loci are independent, the likelihood of the sequence data is a product across all loci. The summation in eq. 8 is over all possible gene tree topologies for the sequences, and the integral is $(n_j - 1)$-dimensional, over the $n_j - 1$ coalescent times on each gene tree. The gene trees and coalescent times are not observed, and the likelihood function averages over them, accommodating their uncertainties.

The species tree S and the MSC parameters Θ can be estimated using ML by maximizing eq. 8. Both the phylogenetic likelihood $f(X_j|G_j, \boldsymbol{t}_j, \eta)$ and MSC density $f(G_j, \boldsymbol{t}_j|S, \Theta)$ are straightforward to calculate, but averaging over all the possible gene tree topologies and coalescent times at each locus is computationally infeasible except for small data sets. The only ML implementation available is the 3S program [44, 45], which enumerates the gene trees and uses numerical integration (Gaussian quadrature) to calculate the integrals. Although limited to three species and three sequences per locus, 3S can handle tens of thousands of loci.

With more than three species, the Bayesian method has a computational advantage over ML, with the Markov chain Monte Carlo (MCMC) algorithm averaging over the gene trees and coalescent times. We assign prior distributions to the species tree and model parameters. For example, the species tree can be assigned a uniform prior over all rooted trees, while the population-size parameters (θs) can be assigned gamma or inverse-gamma priors. The inverse-gamma priors for θs are conjugate (so that both the prior and posterior for θs are inverse-gamma), allowing the θs to be integrated out analytically [51], which helps with MCMC mixing. The age of the species-tree root can be assigned a gamma or inverse-gamma prior, while the other node ages can be constructed using a Dirichlet distribution [52].

The MCMC algorithm samples from the joint posterior distribution of the species tree, the MSC parameters, and the gene trees at all loci

$$f(S, \Theta, \boldsymbol{G}, \boldsymbol{t} | \boldsymbol{X}) \propto f(S, \Theta) \prod_{j=1}^{L} f(X_j | G_j, \boldsymbol{t}_j, \eta) f(G_j, \boldsymbol{t}_j | S, \Theta). \quad (9)$$

In particular the samples of (S, Θ) generated by the algorithm are from the marginal posterior $f(S, \Theta | \boldsymbol{X})$, and the frequency at which a species tree is visited is an estimate of its posterior probability. In this way, MCMC averages out the gene trees and coalescent times numerically.

The first implementation of the Bayesian approach is the program BEST [53]. This uses the samples of gene trees with branch lengths produced by MrBayes [54] and applies an importance-sampling correction because MrBayes does not assume that the gene trees are distributed according to the MSC density. This strategy does not work well, as the species tree and the gene trees place tight constraints on each other in the MSC model. Currently two Bayesian programs under the MSC are in common use: *BEAST [46] and BPP [47–49], both of which explicitly use the MSC model. The algorithm in BPP for species tree inference goes through several proposal steps in each MCMC iteration, as follows.

1. Update the coalescent times $\boldsymbol{t}_j$ on the gene tree at each locus j;
2. Update the gene tree topology G_j at each locus j through a subtree-pruning-and-regrafting (SPR) algorithm;
3. Update the population sizes (θs);
4. Update the species divergence times (τs);
5. Update the species tree topology S through an NNI (nearest-neighbour interchange) or SPR move, which may change the gene trees to avoid conflicts;
6. Use a multiplier to rescale all node ages on the species tree and on all gene trees.

Perhaps the greatest challenge in such MCMC algorithms comes from the constraint between the species tree and the gene trees. Consider step 4 for changing species divergence time τ_{AB}, the age of the ancestral node for two sister species/clades A and B. Let t_{ab} be the sequence divergence time for two sequences from A and B. Then $\tau_{AB} < t_{ab}$. If the dataset includes thousands of loci and many sequences from A and B at each locus, the smallest of t_{ab} among all loci may be almost identical to the current τ_{AB}. Then when we use a sliding window to change τ_{AB}, the window size will have a width near zero, and the MCMC is virtually stuck. A "rubber-band" algorithm was proposed in [8], which changes τ and the affected node ages on gene trees jointly. Similarly, in step 5, it is very inefficient to change the species tree when all gene trees are fixed. A breakthrough was to make coordinated changes to the gene trees when an NNI algorithm is used to change the species tree [47]. The algorithm has since been extended to SPR [48, 55] and ported to *BEAST as well [55, 56]. Those improvements have pushed the limit of datasets that can be analyzed using Bayesian MCMC programs from ∼100 to ∼10,000 loci [19, 20].

Heuristic or summary methods

Many heuristic methods for species tree estimation have been developed, which use summaries of the data rather than the original mutlilocus sequence alignments. For extensive reviews, see [9, 10, 22, 23]. Here we mention four commonly used ones: MP-EST [39], ASTRAL [40], NJ-ST [57], and SVDQuartets [58].

MP-EST [39] estimates triplet gene trees under the molecular clock (rate constancy among lineages), and then uses a composite likelihood function, treating the frequencies of the triplet gene trees as input data from a trinomial distribution (with probabilities given in eq. 4). A composite or pseudo-likelihood is constructed by multiplying those probabilities for all possible triplets, ignoring lack of independence among them. This composite likelihood is maximized to estimate the species tree.

Astral [40] uses a phylogenetic method to infer unrooted gene trees, and extracts the quartets from them. It then finds the species tree that is most compatible with the quartets in the set. A procedure has also been developed to attach local support values for nodes on the inferred species tree [59].

NJ-ST [57] uses a distance method to estimate an unrooted species tree from a collection of unrooted gene trees. The species tree estimate is the neighbor-joining tree built from a distance matrix where the distance between two species is defined as the average number of internal nodes on the gene tree between the species.

All those three methods are two-step methods, treating estimated gene tree topologies as data. They are consistent, with the probability to recover the correct species tree approaching one when the number of gene trees increases. As discussed above, the anomaly zone does not exist for rooted triplets or equivalently for unrooted quartets. However, the argument for consistency is based on the assumption that the input gene trees are known without error. Phylogenetic reconstruction errors are known to affect the performance of two-step methods [60]. Furthermore, as those two-step methods uses gene tree topologies but not branch lengths or coalescent times, they suffer from unidentifiability issues. They can estimate the species tree topology but not all parameters in the MSC model.

Another summary method is called SVDQuartets [58]. This is a quartet method, designed for data of *coalescent-independent sites*, sites that have independent

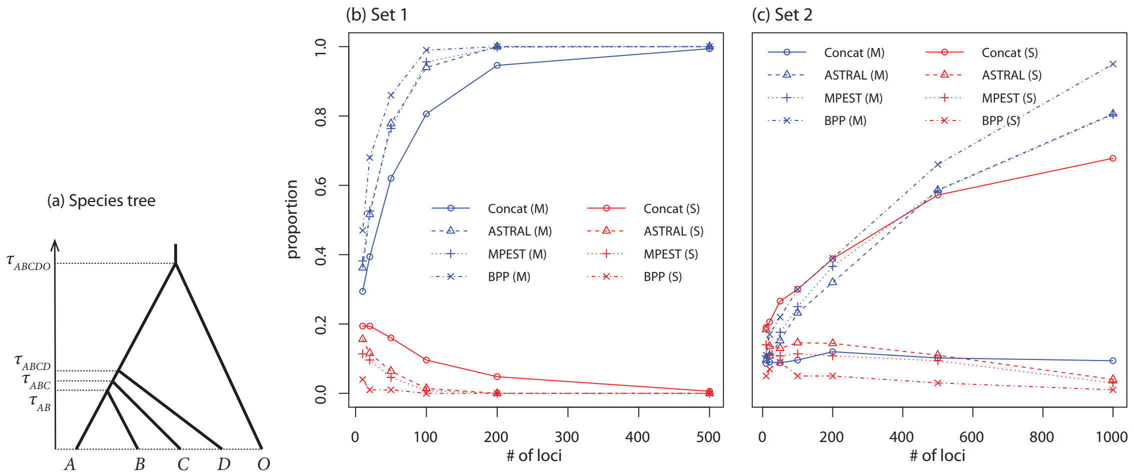

FIG. 5. A simulation experiment to compare four methods of species tree estimation: ML analysis of concatenated data, ASTRAL, MP-EST and BPP. (**a**) Species tree used in the simulation. Two sets of parameter values are used: $\tau_{ABCDO} = 3\theta$, $\tau_{ABCD} = 1.25\theta$, $\tau_{ABC} = 1.125\theta$, and $\tau_{AB} = \theta$ in set 1, and $\tau_{ABCDO} = 3\theta$, $\tau_{ABCD} = 1.05\theta$, $\tau_{ABC} = 1.025\theta$, and $\tau_{AB} = \theta$ in set 2, with $\theta = 0.01$. (**b&c**) Proportion of replicates in which the estimated species tree is the true tree (blue) or the mismatching tree $S = (((A,B),(C,D)),O)$ (red). Data of multi-locus alignments were simulated using the *simulate* option of BPP [49] under the JC69 model [66], with one sequence sampled per species at each locus, and with sequence length to be 500 sites. The outgroup sequence (O) is used to root the tree by concatenation/ML and ASTRAL, but not used by BPP or MP-EST. The number of replicates is 100 for BPP and 500 for the other methods.

histories. Such sites are similar to SNPs but include constant sites as well. Genome sequencing projects do not generate such data. When the method is applied to multilocus sequence alignments, sites are pooled across loci, as in the concatenation method, so that the data are the counts of 256 ($= 4^4$) site patterns for the species quartet. Note that the site-pattern counts pooled across loci are summaries of the original multilocus alignments. When all sites have independent histories, the summation over gene trees and the integral over coalescent times under the MSC model (eq. 8) are analytically tractable [58, 61]. Pooling sites across loci causes information loss and identifiability issues so that the method is unable to identify all parameters in the MSC model even if the species tree topology is identifiable [9, 62].

Some two-step methods use both gene tree topologies and branch lengths (coalescent times) [63]. However, those methods were found to have poorer performance than methods based on topologies alone [64, 65]. This is because the methods ignore random sampling errors in branch-length estimates. It is easy to see that sampling errors in branch lengths may have a major impact on estimation of the species tree and the MSC parameters. For example, if two sequences from two species are identical at a locus so that the estimated coalescent time is $t_{ab} = 0$, the species divergence time τ_{AB} will be forced to be 0 as well (since $\tau_{AB} < t_{ab}$), which may have a dramatic effect on species tree estimation. While coalescent times or branch lengths on gene trees contain much information [62], it is important to accommodate their uncertainties.

Comparison between full-likelihood and heuristic methods

Figure 5 shows results from a small simulation to illustrate the different performance of a full likelihood method (BPP), two summary methods (ASTRAL and MP-EST), and ML analysis of concatenated data. The species tree is challenging with short internal branches in both sets of simulations. BPP recovered the true species tree with higher probability than the two summary methods and concatenation. For set 1, all four methods are consistent, with the probability of recovering the true species tree approaching 1 for every method when the number of loci increases. For set 2 the species tree is in the anomaly zone, and concatenation/ML is inconsistent, with the probability for the mismatching balanced tree approaching 1, while the other three methods are consistent. Note that the ML method applied to concatenated data assumes one tree and one set of divergence times for all loci and can be inconsistent [67].

Heuristic methods based on data summaries have a huge computational advantage over full-likelihood methods. For large datasets with hundreds or thousands of species and thousands of loci, they may be the only methods that are currently feasible computationally. Heuristic methods have poorer statistical performance than full-likelihood methods, and the difference can be large for challenging species trees with short internal branches [9, 19, 62, 64, 68]. As two-step methods typically ignore phylogenetic reconstruction errors in gene trees, their performance may suffer from uncertainties in the gene trees [60, 64]: for those methods, *species trees are only as good as the gene trees on which they are built* [9, 23].

An important strength of full-likelihood methods is that they can provide estimates of parameters in the MSC

model when the species tree is fixed [16, 56]. The MSC model for a species tree of s species has $s-1$ divergence times (τs) and $2s-1$ population sizes (θs) (fig. 2), all of which can be identified and estimated by full likelihood methods using multilocus sequence data. In contrast, summary methods use only a portion of information in the data and are unable to identify all parameters in the model. For example, in the case of three species, the MSC model involves seven parameters (two τs and five θs), but there are only two distinct frequencies of gene trees (eq. 4), so that two-step methods using gene tree topologies alone can identify only the internal branch length in coalescent units: ϕ or $2\Delta\tau/\theta_{BC}$ of eq. 3. For large datasets for which species tree estimation using full-likelihood methods is too expensive, it may be advisable to use summary methods to infer the species tree, and then full likelihood methods to estimate the population parameters on the species tree.

Multispecies Coalescent with Migration or Introgression

In the past two decades, analyses of genomic data have highlighted the prevalence of cross-species gene flow [69–71]. Ancient gene flow has been detected in a variety of species, from mosquitoes [20, 72] and butterflies [73] to hominins [74]. Like deep coalescence, gene flow causes genealogical fluctuations across the genome, posing challenges to species tree estimation [75–78]. Perhaps more importantly, hybridization can lead to rapid genomic changes, leading to beneficial new phenotypes and ecological adaptations. Inferring the mode and timing of gene flow may help us to achieve a better and richer understanding of the process of speciation and adaptation [70, 71].

Two types of models of gene flow have been developed, both as extensions to the MSC model. The first is the migration model (MSC+M), also known as the isolation-with-migration (IM) model [17, 79], which assumes that gene flow occurs at a certain rate every generation. The second is the hybridization/introgression model (MSC+I or MSci) [80, 81], in which hybridization occurs at a fixed time point in the past. Here we discuss the distribution of the gene trees under those models of gene flow. ML and Bayesian methods of inference proceed as before (eqs. 8 & 9), except that the model may involve parameters that measure the timing and strength of gene flow and the gene tree may include the migration or introgression history, as well as the tree topology and coalescent times. We will also mention a few heuristic methods for testing for the presence of gene flow and estimating its rate.

Isolation-with-migration (IM)

Consider two populations A and B with population sizes θ_A and θ_B that have been exchanging migrants at the rates of M_{AB} and M_{BA} since their divergence at time τ_R (fig. 6a). The parameter vector in the IM model for two species is thus $\Theta = \{\theta_A, \theta_B, \theta_R, \tau_R, M_{AB}, M_{BA}\}$. Here the population migration rate $M_{AB} = m_{AB}N_B$ is the expected number of migrants from A to B (in the real world with time running forward) per generation, with m_{AB} to be the proportion of individuals in population B that are immigrants from population A. The rate $M_{BA} = m_{BA}N_A$ is defined similarly. Note that migration rates in the IM model reflect the long-term effects of migration, genetic drift, recombination, as well as natural selection purging introduced alleles [71]. We consider the probability density of the gene trees under the IM model. There are two formulations, depending on whether the gene tree at a locus includes the migration history.

In the first formulation, the gene tree includes the tree topology and coalescent times, but not the migration history (or with the migration history integrated out). This relies on the theory developed in the *structured coalescent* framework in which the backwards-in-time process of coalescence and migration is described using a continuous-time Markov chain [82–84]. The state of the chain is specified by the number of sequences in the sample and their population IDs [18, 45, 61]. Consider the IM model for two species (A and B) of figure 6a and suppose two sequences (a and b) are sampled at locus j (fig. 6b), so that the gene tree is just the sequence divergence time t_j (we suppress the subscript and write t_j as t hence). When we trace the genealogy of the two sequences backwards in time, the sequences may move between populations and they may coalesce. The possible states are s_{AA}, s_{AB}, s_{BB}, s_A and s_B. Here s_{AA} means that both sequences are in population A, s_{BB} means both are in B, while s_{AB} means one is in A and the other in B. With only two sequences in the sample, there is no need to distinguish s_{AB} and s_{BA}. If the two sequences have coalesced, the state becomes s_A or s_B, and these are lumped into one artificial absorbing state, $s_{A|B}$, since there is no need to trace the history any further. Let $Q = \{q_{uv}\}$ be the generator matrix for the Markov chain over the time interval $(0, \tau_R)$, where q_{uv} is the instantaneous rate of transition from states u to v. This is

$$Q = \begin{array}{c|cccc} & s_{AA} & s_{AB} & s_{BB} & s_{A|B} \\ \hline s_{AA} & -2\left(m_1 + \frac{1}{\theta_A}\right) & 2m_1 & 0 & \frac{2}{\theta_A} \\ s_{AB} & m_2 & -(m_1+m_2) & m_1 & 0 \\ s_{BB} & 0 & 2m_2 & -2\left(m_2 + \frac{1}{\theta_B}\right) & \frac{2}{\theta_B} \\ s_{A|B} & 0 & 0 & 0 & 0 \end{array} \qquad (10)$$

Here the time unit is one mutation per site, $m_1 = \frac{4M_{BA}}{\theta_A} = \frac{m_{BA}}{\mu}$ is the *mutation-scaled migration rate* into species A and $m_2 = \frac{4M_{AB}}{\theta_B} = \frac{m_{AB}}{\mu}$ is the rate into B. Note that the Markov chain runs backwards in time while the migration rates (e.g., M_{AB} and m_2) are defined under the real-world forward-in-time view. For example, in the first row, the transition from s_{AA} to s_{AB} represents migration from B to A in the real world, and either sequence in A can be the migrant, so that the rate is $2m_{BA}$ per generation or $2m_{BA}/\mu = 2m_1$ per mutational

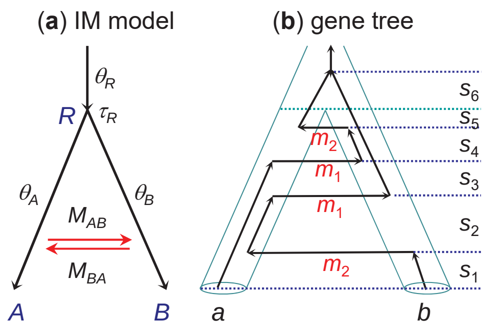

FIG. 6. (**a**) Migration (MSC+M) or isolation-with-migration (IM) model for two species (A and B) showing the parameters. (**b**) A gene tree for two sequences (a and b) with divergence time t and four migration events, with $t = \sum_{k=1}^{6} s_k$. The migration rates (per mutational time unit) are shown beneath the horizontal lines representing migration events. Note that time runs forward in (**a**) when we define migration rates (M_{AB} or m_2) and backward in (**b**) when we trace the genealogical history at the locus.

time unit. The transition from s_{AA} to $s_{A|B}$ means that the two sequences coalesce in A, with rate $\frac{2}{\theta_A}$. State s_{BB} is not reachable from s_{AA} instantaneously.

The transition probability matrix over any time $0 < t < \tau_R \equiv \tau$ is then $P(t) = \{p_{uv}(t)\} = \mathrm{e}^{Qt}$, where $p_{uv}(t)$ is the probability that given state u at time 0, the chain will be in state v at time t. $P(t)$ is analytically tractable in special cases (e.g., when the model is symmetrical with $M_{AB} = M_{BA}$ and $\theta_A = \theta_B$, [18]) but can be calculated in general using efficient algorithms for matrix exponentiation. Let s_0 be the initial state, which is one of s_{AA}, s_{AB}, and s_{BB}, depending on which species each sequence is sampled from ($s_0 = s_{AB}$ in the gene tree of fig. 6b). The density of the divergence time t is

$$f(t|\Theta) = \begin{cases} p_{s_0 s_{AA}}(t)\,\frac{2}{\theta_A} + p_{s_0 s_{BB}}(t)\,\frac{2}{\theta_B}, & \text{if } t < \tau, \\ \left[1 - p_{s_0 s_{A|B}}(\tau)\right] \frac{2}{\theta_R}\,\mathrm{e}^{-\frac{2}{\theta_R}(t-\tau)}, & \text{if } t \geq \tau. \end{cases} \tag{11}$$

Recall that the probability density $f(t)$ means that $f(t)\Delta t$ is the probability that the divergence time is in the small interval $(t, t+\Delta t)$. In the case of $t < \tau$, the two sequences coalesce before reaching τ. The probability $f(t)\Delta t$ is a sum of two terms, corresponding to the coalescent occurring in either A or B. The first term, $p_{s_0 s_{AA}}(t)\frac{2}{\theta_A}\Delta t$, is the probability that both sequences are in species A right at t, times the probability, $\frac{2}{\theta_A}\Delta t$, that they coalesce during $(t, t+\Delta t)$. Similarly the second term is the probability of coalescent occurring in B. In the case of $t > \tau$, the two sequences do not coalesce in either A or B before time τ and both enter the ancestral species R. Here $1 - p_{s_0 s_{A|B}}(\tau)$ is the probability that the Markov chain is in any of the two-sequence states at time τ (in other words, sequences a and b have not coalesced by time τ). Inside species R, the two sequences coalesce at the rate $\frac{2}{\theta_R}$, with the waiting time $(t-\tau)$ exponentially distributed.

Note that calculation of $P(t)$ for the Markov chain integrates out the migration history at each locus analytically, so that eq. 11 is a function of divergence time t but not of the migration events or times. Even in the case of two sequences (fig. 6b), there are an infinite number of migration histories that give rise to the same t, and eq. 11 averages over all of them.

The Markov chain (Q) specified above applies to two species and two sequences. A different Markov chain has to be constructed if there are more species or more sequences. The theory is general and works for arbitrary numbers of species and sequences. For a tree of s extant species, we divide the timeline into s epochs according to the $(s-1)$ species divergence times. In each epoch, the populations are fixed so that the coalescent and migration rates stay the same, and a Markov chain can be constructed [18, 61]. With the MSC density of gene trees calculated this way, the likelihood under the IM model is given by eq. 8, although the parameter vector Θ includes the migration rates as well. This strategy of integrating out the migration history may offer a huge computational advantage. However, the number of states in the Markov chain grows explosively with the increase in the number of species and the number of sequences [61]. The formulation is feasible for very small numbers of species and sequences only. The only implementation of this strategy appears to be the ML program 3s [18, 45], which is limited to three species and three sequences, although tens of thousands of loci can be handled.

In the second formulation, the gene tree at a locus includes the tree topology, coalescent times, and the full migration history including the number, times and directions of migration events (fig. 6b). The probability density for such a gene tree is easy to compute because both coalescent and migration are Poisson events with exponential waiting times [85–87]. In the gene tree of figure 6b, the time period $(0,t)$ is broken into six time segments by the coalescent, migration, and speciation events, and within each segment, the number of lineages is constant, as are the coalescent and migration rates. Then the probability density of the gene tree (G) is given by the rates for the coalescent and migration events times the probability of no events over the whole time period

$$f(G|\Theta) = \left[m_1^2\,\mathrm{e}^{-\frac{2}{\theta_A}s_2 - m_1(s_1+2s_2+s_3+s_5)}\right] \times \left[m_2^2\,\mathrm{e}^{-\frac{2}{\theta_B}s_4 - m_2(s_1+s_3+2s_4+s_5)}\right] \times \left[\frac{2}{\theta_R}\,\mathrm{e}^{-\frac{2}{\theta_R}(t-\tau)}\right]. \tag{12}$$

The three pairs of brackets represent contributions to the gene-tree density from species A, B, and R, respectively. For species A, there are 2 migration events into A (with rates m_1^2), coalescent does not occur over time segment s_2, and migration does not occur over segments s_1, s_2, s_3, or s_5, during which the number of lineages is 1, 2, 1, and 1, respectively. Hence the term for species A. Note that the probability of no events, or the probability that none of multiple independent Poisson events with a total rate of λ occurs, over time t is $\mathrm{e}^{-\lambda t}$. The contribution from species B is given similarly. In species R, a coalescent

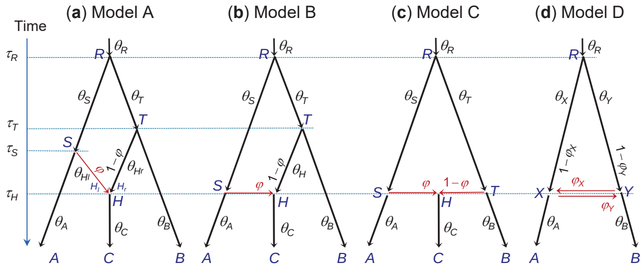

FIG. 7. (**a**) MSci models A, B, C, and D implemented in BPP [81], showing the parameters. In model A, two parental species SH and TH merge to form a hybrid species H at time τ_H, but both parental species become extinct (see fig. 8a&b for alternative interpretations). In model B, there is introgression from species RA to TC at time $\tau_S = \tau_H$. In model C, species RA and RB come into contact to form hybrid species HC at time $\tau_S = \tau_H = \tau_T$. Model D assumes bidirectional introgression between species RA and RB at time $\tau_X = \tau_Y$. Here the introgression probability (φ) is assigned to the horizontal (introgression) branch at each hybridization node whereas in [81] it is sometimes assigned to the vertical branch.

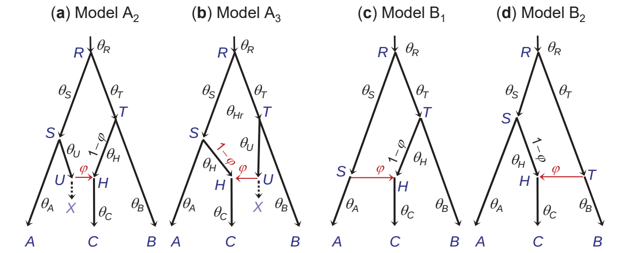

FIG. 8. (**a&b**) Two interpretations of model A, alternative to figure 7a, involving a ghost species X. In model A$_2$, species SUX contributes migrants to species THC at time τ_H and has since become extinct or unsampled in the data, while in model A$_3$, TUX is the ghost species. Models A$_1$ (fig. 7a), A$_2$, and A$_3$ are indistinguishable using genetic data. (**c&d**) Two versions of model B, which are identifiable using genetic data.

occurs after the waiting time $s_6 = t - \tau$, so the rate is $\frac{2}{\theta_R}$ and the probability of no event is $\mathrm{e}^{-\frac{2}{\theta_R}(t-\tau)}$.

Unlike eq. 11 in which the gene tree means divergence time t, here G represents the full coalescent and migration history at the locus, such as the (backwards-in-time) transitions of sequence b from B into A at time s_1 and back to B at time $s_1 + s_2$, and so on. If we sum over all possible histories which have divergence time t (one of which is that of fig. 6b), the marginal density $f(t)$ will be given by eq. 11.

Eq. 12 is easily generalizable to more species and sequences. For a general gene tree, one can break the time period from the present time to the root of the gene tree into time segments by the coalescent and migration events at the locus and by the speciation events. Then the probability density of the gene tree is simply given as the product of rates for the coalescent and migration events that occurred times the probability of no events over the whole time period.

This formulation is used in Bayesian implementations of the IM model such as IMA [88, 89] and G-PHOCS [90]. The posterior is given by eq. 9 except that the gene tree G_j includes the migration history. G-PHOCS is an extension of an earlier version of BPP [8, 16] and is computationally more efficient than IMA and can deal with a few thousand loci. The algorithm averages over the migration history at every locus and becomes inefficient at high migration rates, as there will be many migration events to average over. Note that the sequence likelihood depends on the gene tree and coalescent times but not migration events.

Multispecies coalescent with introgression (MSci)

The introgression or MSci model assumes that gene flow occurs between species at fixed time points in the past (fig. 7). There are two types of nodes on the species tree: speciation nodes and hybridization nodes. While a speciation node (if it is not the root) has one parent, a hybridization node has two parents, with their contributions to the hybrid species represented by probabilities φ and $1 - \varphi$. When we trace the history of sequences backwards in time and meet a hybridization node, each sequence picks one of the two parents according to probabilities φ and $1 - \varphi$. The parameters in the model includes the introgression probabilities as well as the species divergence/introgression times (τs) and population sizes (θs), with $\Theta = \{\boldsymbol{\tau}, \boldsymbol{\theta}, \boldsymbol{\varphi}\}$. The introgression probability φ, also written as γ, has been called (inappropriately) ‘inheritance probability’ or ‘heritability’. Like the migration rate in the IM model, the introgression probability reflects the long-term effects of drift and selection on introgressed alleles. The MSci model has been referred to as the network multispecies coalescent (NMSC) [91, 92] or multispecies network coalescent (MSNC) [93, 94]. We avoid the term ‘network’ as it has been used to refer to a variety of processes including gene-tree reconstruction errors [95].

Four types of MSci models are implemented in BPP (fig. 7) [81]. In model A, two species SH and TH merge to form a hybrid species HC. This scenario may be rare, but the model can be used to accommodate introgressions involving ghost or unsampled species (fig. 8a&b). Model B assumes introgression from species RA to TC at time $\tau_S = \tau_H$. This is distinguishable using genetic data from the alternative model in which there is introgression from RB to SC (B$_2$, fig. 8d). Model C (fig. 7c) is a case of hybrid speciation. Model D assumes that two species RA and RB came into contact at time $\tau_X = \tau_Y$ and exchanged migrants.

The two parental branches are sometimes called the ‘major hybrid edges’ and ‘minor hybrid edges’, according as $\varphi > \frac{1}{2}$, and the binary species tree that remains after all minor hybrid branches are removed is called the ‘major species tree’ [95]. This characterization is useful if gene flow occurs in pulses as assumed by the MSci model but may be misleading if gene flow is continuous. For example, continuous migration at a low rate per generation can drastically change the gene tree distribution so that when the MSci model is fitted to the data, the major species tree may reflect gene flow, rather than species divergences [20, 72, 78].

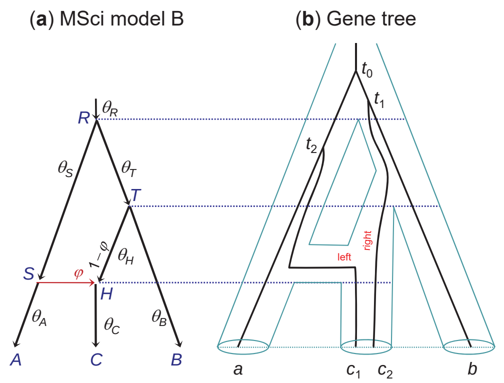

FIG. 9. (**a**) MSci model B for three species (fig. 7b) and (**b**) a gene tree for four sequences for illustrating the gene-tree density under the MSci model.

Below we consider the probabilities of gene tree topologies under the MSci model. These can be used in the two-step methods to estimate the introgression probabilities or to infer the introgression model using reconstructed gene trees as input data, as in the PHYLONET/ML program [96].

The calculation is very similar to that under the simple MSC model (eq. 4). Consider model B (fig. 9a), with three sequences at the locus (a,b,c) [78]. If sequences b and c coalesce in species T, the gene tree will be $G_1 = (a,(b,c))$, while if a and c coalesce in species S, the gene tree will be $G_2 = (b,(c,a))$. If neither event occurs, the two coalescent events for the three sequences will occur in species R and the three gene trees will occur with equal probabilities. Thus $G_3 = (c,(a,b))$ must be the least probable gene tree. We have

$$\begin{aligned} \mathbb{P}(G_1) &= \tfrac{1}{3}\varphi\phi_S + (1-\varphi)\left(1-\phi_T+\tfrac{1}{3}\phi_T\right), \\ \mathbb{P}(G_2) &= \varphi\left(1-\phi_S+\tfrac{1}{3}\phi_S\right)+\tfrac{1}{3}(1-\varphi)\phi_T, \\ \mathbb{P}(G_3) &= \tfrac{1}{3}\left[\varphi\phi_S+(1-\varphi)\phi_T\right] = 1-\mathbb{P}(G_1)-\mathbb{P}(G_2), \end{aligned} \tag{13}$$

where $\phi_S = \mathrm{e}^{-\frac{2}{\theta_S}(\tau_R-\tau_S)}$ and $\phi_T = \mathrm{e}^{-\frac{2}{\theta_T}(\tau_R-\tau_T)}$ are the probabilities that two sequences entering species S or T do not coalesce in that species (cf. ϕ of eq. 3). Consider gene tree G_1, which means that sequences b and c coalesce first. If sequence c enters S (which happens with probability φ), G_1 can occur only if sequences c and a do not coalesce in S. Hence the first term, $\varphi\phi_S \cdot \frac{1}{3}$. If sequence c enters H (which happens with probability $1-\varphi$), sequences b and c can coalesce in T or R. Hence the second term, $(1-\varphi)\left(1-\phi_T+\frac{1}{3}\phi_T\right)$.

The gene tree probabilities (eq. 13) are functions of φ, ϕ_S and ϕ_T, while ϕ_S and ϕ_T are simple functions of the internal branch lengths in coalescent units on the species tree. We have $\mathbb{P}(G_1) < \mathbb{P}(G_2)$ if $(1-\varphi)(1-\phi_T) < \varphi(1-\phi_S)$, or if b and c are more likely to coalesce in T than are a and c to coalesce in S [78].

Next we consider the joint density of $(G_j, \boldsymbol{t}_j)$, the gene tree with the complete history of coalescence and introgression events at locus j, including the parental path taken by each sequence at each hybridization node. This is used in full-likelihood implementations of the MSci model. This joint density is very similar to that under the MSC without gene flow (eq. 7), with the only modification that each time a sequence passes a hybridization node, there is a probability φ or $1-\varphi$ depending on the parental path taken. Thus

$$\begin{aligned} f(G_j, \boldsymbol{t}_j | S, \Theta) = &\left[\mathrm{e}^{-\frac{2}{\theta_C}\tau_H}\right] \times \left[\varphi\frac{2}{\theta_S}\mathrm{e}^{-\frac{2}{\theta_S}(t_2-\tau_S)}\right] \\ &\times \left[1-\varphi\right] \times \left[\frac{2}{\theta_T}\mathrm{e}^{-\frac{2}{\theta_T}(\tau_R-\tau_T)}\right] \\ &\times \left[\frac{2}{\theta_R}\cdot\frac{2}{\theta_R}\mathrm{e}^{-\frac{6}{\theta_R}(t_1-\tau_R)-\frac{2}{\theta_R}(t_0-t_1)}\right]. \end{aligned} \tag{14}$$

The five pairs of brackets correspond to species C, S, H, T and R (fig. 9b). For species S (i.e., SR), sequence c_1 picks parental path S and coalesces with sequence a at time t_2, so that the contribution to the gene-tree density from S is $\varphi\frac{2}{\theta_S}\mathrm{e}^{-\frac{2}{\theta_S}(t_2-\tau_S)}$. Introgression is counted as an event in the receiving population (rather than the source population) when we trace the lineages backwards in time and reach a hybridization node.

Bayesian implementations of the introgression model can then proceed as before, with the joint posterior of the MSci model and parameters given by eq. 9, except that S now represents the MSci model, the parameter vector Θ includes the introgression probabilities (φs) as well as the divergence/introgression times (τs) and population sizes (θs), and the gene tree G_j includes the introgression history at the locus. There are currently three Bayesian MCMC implementations of the MSci model: PHYLONET/MCMC-SEQ [93], *BEAST [94, 97], and BPP [81] (table 1). PHYLONET and *BEAST can allow changes to hybridization events in the MCMC and can infer the introgression model from the data. Those programs appear to reach their limits with ~ 100 loci. BPP assumes that the MSci model is specified and fixed and the program estimates the parameters under the model. It has been applied to datasets of over 10,000 loci [29, 81]. Also BPP implements four different types of introgression models (fig. 7), while only model A is available in PHYLONET and *BEAST.

Binary species trees generated by taking different parental paths at hybridization nodes are called “displayed species trees” [92] or “parental species trees”. An interesting formulation of the MSci model specifies the distribution of the gene trees as a mixture over the displayed species trees, with the mixing probabilities given by the introgression probabilities at the hybridization nodes (fig. 10) [e.g., 98, 99]. To simulate a gene tree, one would sample a displayed species tree first and then generate the gene tree according to the simple MSC model. This is in general incorrect as it forces all sequences at the locus to take the same parental path at each hybridization node, whereas correctly there should be a binomial sampling process when two or more sequences reach a hybridization node. In the model of figure 10, if sequences b and c reach species Y, it should be possible

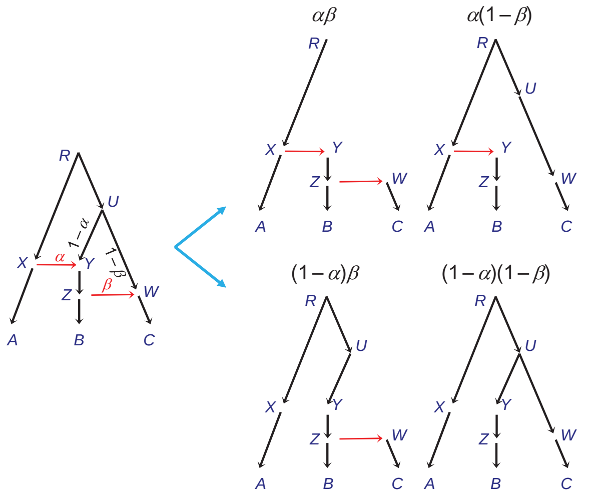

FIG. 10. Displayed species trees are binary trees that result from removing one of the two parental branches at each hybridization node in the MSci model. With k hybridization nodes, there are 2^k displayed species trees. Their probabilities are given by the introgression probabilities at the hybridization nodes: $\alpha\beta$, $\alpha(1-\beta)$, $(1-\alpha)\beta$, and $(1-\alpha)(1-\beta)$.

for one of them to take the left parent and the other the right parent. In the special case where each hybridization node on the species tree has at most one sequence from all its descendant populations, the formulation is correct and can be used to derive the probability distribution of gene trees. For example, eq. 13 for the case of three species and three sequences (fig. 9a) can be derived this way. It is also interesting to note that under the MSci model, the most probable gene tree may have a topology that is different from all of the displayed species trees [100].

Heuristic methods for inferring gene flow

A number of heuristic methods have been developed to test for the presence of gene flow and to estimate its strength. Here we mention a few briefly. The most popular method is the D-statistic or *ABBA*-*BABA* test [101]. This uses the species tree $(((A,B),C),O)$ for three species A, B, and C, with the outgroup species O, and is based on the counts of site patterns when one sequence or genome is available from each species [102]. There are three parsimony-informative site patterns: *AABB* matches the species tree, while *ABBA* and *BABA* are the mismatching patterns, where A and B are any two distinct nucleotides. The probabilities for the two mismatching site patterns *ABBA* and *BABA* should be equal if there exists deep coalescence but no gene flow, but they are different if there is gene flow between the non-sister species (A and C or B and C) in addition to deep coalescence. Thus gene flow can be tested by using the site-pattern frequencies to examine the deviation of

$$D = \frac{f_{ABBA} - f_{BABA}}{f_{ABBA} + f_{BABA}} \tag{15}$$

from 0. The D-statistic has been extended to the case of five species, assuming a symmetric species tree in the so-called D_{FOIL} test [103]. The site pattern frequencies can also be used to estimate the introgression probability, as in the program HyDe [104, 105]. From

$$\frac{f_1}{f_2} = \frac{p_{AABB} - p_{ABAB}}{p_{ABBA} - p_{ABAB}} = \frac{\varphi}{1-\varphi}, \tag{16}$$

one gets the estimate

$$\hat{\varphi} = \frac{\hat{f}_1}{\hat{f}_1 + \hat{f}_2}. \tag{17}$$

This is based on the hybridization model with $\tau_S = \tau_T$ and $\theta_S = \theta_T$ (fig. 7c). The estimate should be biased if this symmetry does not hold.

A similar argument may be applied to gene tree topologies instead of site patterns. The probabilities of the two mismatching gene trees $((b,c),a)$ and $((c,a),b)$ are equal if there exists deep coalescence but no gene flow, but different if there is in addition gene flow between the non-sister species (A and C or B and C). Thus the observed frequencies of gene tree topologies can be used to estimate the introgression probability, as in the SNaQ method [95, 106]. Assume $\phi_S = \phi_T = \phi$ in eq. 13 and let $f_2 = \mathbb{P}(G_2) = \frac{1}{3}\phi + \varphi(1-\phi)$ and $f_3 = \mathbb{P}(G_3) = \frac{1}{3}\phi$ be the probabilities of the two mismatching gene trees. Then

$$\hat{\varphi} = \frac{\hat{f}_2 - \hat{f}_3}{1 - \hat{f}_3}. \tag{18}$$

The D-statistic cannot be used to detect gene flow between sister species or to estimate the time of introgression. Such unidentifiability issues also exist in other methods which detect hybridization events using genome-wide averages, such as the average interspecies sequence divergence [107] or the joint allele frequency spectrum [108].

Table 1. A partial list of computer programs implementing the MSC model with and without gene flow

Method	MSC	IM & MSci
Full likelihood	3s	3s
	BPP	IMa3
	*BEAST	G-PhoCS
		BPP
		*BEAST
		PhyloNet
Two-step	ASTRAL	PhyloNet
	MP-EST	PhyloNetworks
	NJ-st	

Unidentifiability, low information content, and challenges of identifying the mode of gene flow

One area where more research is urgently needed is the identifiability of introgression models. If the probability distributions of the data are identical for two sets of parameter values (Θ and Θ'), with $f(X|\Theta) = f(X|\Theta')$ for essentially every dataset X, then Θ is unidentifiable given data X. Several studies have examined identifiability issues of summary methods that use gene tree topologies as data [76, 80, 91, 109], but little research has been done on full likelihood methods.

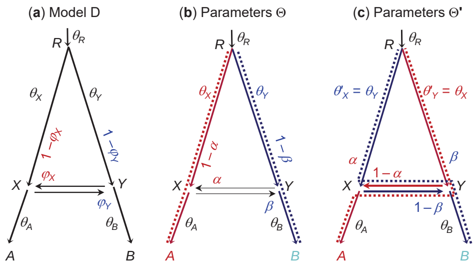

FIG. 11. MSci model D (bidirectional introgression) (fig. 7d) has an identifiability issue. (**a**) Model D showing the definitions of parameters. (**b** & **c**) Two sets of parameter values Θ and Θ' that are unidentifiable. The dotted lines indicate the main routes taken by sequences sampled from species A and B, if the introgression probabilities α and β are $< \frac{1}{2}$.

Some cases of unidentifiability are easy to identify. If it is impossible for two or more sequences to be in one species when we trace the genealogical history of the sample backwards in time, the population size (θ) for that species will be unidentifiable, since it takes two sequences to define a distance. For example, in the MSC model with no gene flow (fig. 2), the population sizes for the extant species are unidentifiable if only one sequence is sampled from each species per locus, but this unidentifiability disappears when multiple sequences are available from each species. Furthermore parameters or models that are unidentifiable using gene tree topologies alone may become identifiable when both gene trees and branch lengths (coalescent times) are used. In the case of three species, there are only three gene trees, so that use of gene tree topologies can identify only one (under the MSC model) or two (under the MSci model) parameters, whereas there are 7 (fig. 2) and 13 (fig. 7a) parameters in those two models respectively, which are all identifiable when information from both gene trees and coalescent times is used.

The identifiability of full-likelihood methods applied to data of multilocus sequence alignments, with multiple sequences per species, is the most interesting case, because full-likelihood methods are expected to be optimal from a statistical point of view and because multilocus alignments are the dominating data form in such analyses. Flouri et al. [81] conjectured that the MSci model is identifiable on multilocus sequence alignments as long as it is identifiable on data of gene trees with coalescent times. Given this, the problem of identifiability can be studied by considering the gene trees with coalescent times (G_j and $\boldsymbol{t}_j$) as the input data.

It is noted that MSci model D (fig. 7d) has an unidentifiability issue of the label-switching type [81] (fig. 11). For every set of parameters, $\Theta = (\theta_R, \theta_A, \theta_B, \theta_X, \theta_Y, \tau_R, \tau_X, \varphi_X, \varphi_Y)$, there is a "mirror" point Θ', which has identical parameter values as Θ except that $\theta'_X = \theta_Y$, $\theta'_Y = \theta_X$, $\varphi'_X = 1 - \varphi_X$ and $\varphi'_Y = 1 - \varphi_Y$. Both Θ and Θ' have exactly the same likelihood, $f(X|S,\Theta) = f(X|S,\Theta')$ for all possible data X. This is a label-switching issue, and does not affect the utility of the model: one may apply a constraint such as $\varphi_X > \frac{1}{2}$ to remove the unidentifiability or apply more sophisticated post-processing of the MCMC sample if the "twin towers" are not well separated [110]. The cases where the bidirectional introgression involves non-sister species or where there are multiple introgression events are yet to be studied.

Even if all parameters are identifiable, typical datasets may lack information for their reliable estimation. For example, typical datasets may be highly informative about species divergence times, but not about population sizes for ancestral species, especially if those species correspond to very short branches on the species tree [111]. In the case of three species both gene flow between non-sister species and population structure in the ancestral species can cause the asymmetry in the probabilities of the two mismatching gene trees [112], so that the two models are unidentifiable using gene tree topologies alone. In general it may be hard to distinguish the different models of gene flow, such as the complete isolation model (MSC with no gene flow), the migration (IM) model, the isolation-with-initial-migration (IIM) model [113], and the introgression (MSci) model. Simulation may be useful to evaluate the power to distinguish such models using genomic datasets.

Conclusion

The multispecies coalescent model provides a powerful framework for analysis of genomic sequences sampled from multiple species to extract the rich information about the evolutionary history of the species. Incorporating species phylogeny in population genetic models of population subdivision opens up opportunities for addressing many exciting questions in evolutionary biology, such as detecting gene flow during and after species formation and delineating species boundaries, as well as inferring demographic changes and estimating population sizes for extinct ancestral species. As discussed in [92], the basic MSC model accommodating species phylogeny and coalescent is in effect a null model, which can be extended to include other important biological processes, leading to models such as

- H_0 : MSC (null model)
- H_1 : MSC + migration (MSC+M or IM model)
- H_2 : MSC + introgression (MSC+I or MSci model)
- H_3 : MSC + population structure
- H_4 : MSC + recombination
- etc.

Currently, large differences exist between full-likelihood methods and heuristic methods. The former have higher statistical efficiency while the latter are orders-of-magnitude faster computationally. There is thus much room for improvement for both classes of methods. For the present, a pragmatic approach to analyzing large datasets may be to use summary methods

to estimate the species tree and then full likelihood methods to estimate the parameters.

Analysis of the simple three-species case [62] suggests that there is rich historical information both in gene-tree branch lengths (which 2-step methods such as ASTRAL, MP-EST, and SNAQ ignore) and in the stochastic fluctuation of genealogical history across loci (which genome-averaging approaches such as SVDQUARTETS and D-statistic ignore). Heuristic methods that make use of both kinds of information may thus have much improved power. For Bayesian implementations of the MSC model, mixing inefficiency of the MCMC algorithm appears to be a far more serious problem than the increase in computational cost for each MCMC iteration [48]. Developing smart proposal algorithms that respect and accommodate the mutual constraints between the species tree and the gene trees is likely to bring dramatic improvement to the capacity of the full-likelihood methods. To empirical biologists the MSC framework makes it possible to ask exciting evolutionary questions; to method developers it offers rich opportunities for testing cutting-edge algorithms in computational statistics (in particular, trans-model MCMC algorithms). With the advancements of sequencing technologies and rapid accumulation of genomic sequence data as the driving force, the field will in all likelihood continue to be a research hotspot in the coming years.

Acknowledgments

This study has been supported by Biotechnology and Biological Sciences Research Council grants (BB/P006493/1 and BB/T003502/1) to Z.Y.

References

[1] J.F.C. Kingman. The coalescent. *Stochastic Processes Appl.*, 13:235–248, 1982.

[2] R.R. Hudson. Testing the constant-rate neutral allele model with protein sequence data. *Evolution*, 37:203–217, 1983.

[3] F. Tajima. Evolutionary relationship of DNA sequences in finite populations. *Genetics*, 105(2):437–460, 1983.

[4] R.R. Hudson. Gene genealogies and the coalescent process. In D J. Futuyma and J.D. Antonovics, editors, *Oxford Surveys in Evolutionary Biology*, pages 1–44. Oxford University Press, New York, 1990.

[5] J. Hein, M.H. Schieriup, and C. Wiuf. *Gene Genealogies, Variation and Evolution: A Primer in Coalescent Theory*. Oxford University Press, Oxford, 2005.

[6] M. Nordborg. Coalescent theory. In D. Balding, M. Bishop, and C. Cannings, editors, *Handbook of Statistical Genetics*, pages 843–877. Wiley, San Francisco, 2007.

[7] J. Wakeley. *Coalescent Theory: An Introduction*. Roberts & Company, Greenwood Village, Colorado, 2009.

[8] B. Rannala and Z. Yang. Bayes estimation of species divergence times and ancestral population sizes using DNA sequences from multiple loci. *Genetics*, 164(4):1645–1656, 2003.

[9] B. Xu and Z. Yang. Challenges in species tree estimation under the multispecies coalescent model. *Genetics*, 204(4): 1353–1368, 2016.

[10] L. Kubatko. The multispecies coalescent. In D. Balding, I. Moltke, and J. Marioni, editors, *Handbook of Statistical Genomics*, pages 219–245. Wiley, New York, 4th edition, 2019.

[11] B. Rannala, S. Edwards, A.D. Leaché, and Z. Yang. The multispecies coalescent model and species tree inference. In C. Scornavacca, F. Delsuc, and N. Galtier, editors, *Phylogenetics in Genomic Era,* book section 3.3, pages 3.3:1–20. No Commercial Publisher, 2020.

[12] B. Rannala and Z. Yang. Species delimitation. In C. Scornavacca, F. Delsuc, and N. Galtier, editors, *Phylogenetics in Genomic Era,* book section 5.5, pages 5.5:1–18. No Commercial Publisher, 2020.

[13] S.V. Edwards. Is a new and general theory of molecular systematics emerging? *Evolution*, 63(1):1–19, 2009.

[14] J.H. Gillespie and C.H. Langley. Are evolutionary rates really variable. *J. Mol. Evol.*, 13:27–34, 1979.

[15] N. Takahata. An attempt to estimate the effective size of the ancestral species common to two extant species from which homologous genes are sequenced. *Genet. Res.*, 48: 187–190, 1986.

[16] R. Burgess and Z. Yang. Estimation of hominoid ancestral population sizes under bayesian coalescent models incorporating mutation rate variation and sequencing errors. *Mol. Biol. Evol.*, 25:1979–1994, 2008.

[17] J. Hey and R. Nielsen. Multilocus methods for estimating population sizes, migration rates and divergence time, with applications to the divergence of *Drosophila pseudoobscura* and *D. persimilis*. *Genetics*, 167:747–760, 2004.

[18] T. Zhu and Z. Yang. Maximum likelihood implementation of an isolation-with-migration model with three species for testing speciation with gene flow. *Mol. Biol. Evol.*, 29: 3131–3142, 2012.

[19] C.M. Shi and Z. Yang. Coalescent-based analyses of genomic sequence data provide a robust resolution of phylogenetic relationships among major groups of gibbons. *Mol. Biol. Evol.*, 35:159–179, 2018.

[20] Y. Thawornwattana, D.A. Dalquen, and Z. Yang. Coalescent analysis of phylogenomic data confidently resolves the species relationships in the *Anopheles gambiae* species complex. *Mol. Biol. Evol.*, 35(10):2512–2527, 2018.

[21] J.H. Degnan and N.A. Rosenberg. Gene tree discordance, phylogenetic inference and the multispecies coalescent. *Trends Ecol. Evol.*, 24:332–340, 2009.

[22] Z. Yang. *Molecular Evolution A Statistical Approach*. Oxford University Press, Oxford, England, 2014.

[23] L. Liu, Z. Xi, S. Wu, C. Davis, and S.V. Edwards. Estimating phylogenetic trees from genome-scale data. *Ann. NY. Acad. Sci.*, page doi: 10.1111/nyas.12747, 2015.

[24] S. Sankararaman. Methods for detecting introgressed archaic sequences. *Curr. Opin. Genet. Dev.*, 62:85–90, 2020. doi: 10.1016/j.gde.2020.05.026.

[25] K. L. Korunes and A. Goldberg. Human genetic admixture. *PLoS Genet.*, 17(3):e1009374, 2021. doi: 10.1371/journal.pgen.1009374.

[26] R. Fisher. The distribution of gene ratios for rare mutations. *Proc. R. Soc. Edin.*, 50:205–220, 1930.

[27] S. Wright. Evolution in Mendelian populations. *Genetics*, 16:97–159, 1931.

[28] N. Yu, F. C. Chen, S. Ota, L. B. Jorde, P. Pamilo, L. Patthy, M. Ramsay, T. Jenkins, S. K. Shyue, and W. H. Li. Larger genetic differences within africans than between africans and eurasians. *Genetics*, 161:269–274, 2002.

[29] Y. Thawornwattana, J. Mallet, and Z. Yang. Complex introgression history of the erato-sara clade of Heliconius butterflies. *bioRxiv*, 2021. doi: 10.1101/2021.02.10.430600.

[30] A.W.F. Edwards. Estimation of the branch points of a branching diffusion process. *J. R. Stat. Soc. B*, 32(2): 155–164, 1970.

[31] N. Takahata. Gene genealogy in three related populations: consistency probability between gene and population trees. *Genetics*, 122:957–966, 1989.

[32] L. Liu and S.V. Edwards. Phylogenetic analysis in the anomaly zone. *Syst. Biol.*, 58(4):452–460, 2009.

[33] J.H. Degnan and L.A. Salter. Gene tree distributions under the coalescent process. *Evolution*, 59:24–37, 2005.

[34] J.H. Degnan and N.A. Rosenberg. Discordance of species trees with their most likely gene trees. *PLoS Genetics*, 2 (5):e68, 2006.

[35] P. Pamilo and M. Nei. Relationships between gene trees and species trees. *Mol. Biol. Evol.*, 5(5):568–583, 1988.

[36] C.W. Linkem, V.N. Minin, and A.D. Leaché. Detecting the anomaly zone in species trees and evidence for a misleading signal in higher-level skink phylogeny (squamata: Scincidae). *Syst. Biol.*, 65(3):465–477, 2016.

[37] A. Cloutier, T.B. Sackton, P. Grayson, M. Clamp, A.J. Baker, and S.V. Edwards. Whole-genome analyses resolve the phylogeny of flightless birds (Palaeognathae) in the presence of an empirical anomaly zone. *Syst. Biol.*, 68: 937–955, 2019.

[38] Y. Wu. Coalescent-based species tree inference from gene tree topologies under incomplete lineage sorting by maximum likelihood. *Evolution*, 66:763–775, 2012.

[39] L. Liu, L. Yu, and S.V. Edwards. A maximum pseudo-likelihood approach for estimating species trees under the coalescent model. *BMC Evol. Biol.*, 10(1):302, 2010.

[40] S. Mirarab and T. Warnow. ASTRAL-II: coalescent-based species tree estimation with many hundreds of taxa and thousands of genes. *Bioinformatics*, 31(12):i44–i52, 2015.

[41] W.P. Maddison. Gene trees in species trees. *Syst. Biol.*, 46 (3):523–536, 1997.

[42] R. Nichols. Gene trees and species trees are not the same. *Trends Ecol. Evol.*, 16:358–364, 2001.

[43] S. V. Edwards, W. B. Jennings, and A. M. Shedlock. Phylogenetics of modern birds in the era of genomics. *Proc. R. Soc. B.*, 272:979–992, 2005.

[44] Z. Yang. Likelihood and Bayes estimation of ancestral population sizes in hominoids using data from multiple loci. *Genetics*, 162(4):1811–1823, 2002.

[45] D. Dalquen, T. Zhu, and Z. Yang. Maximum likelihood implementation of an isolation-with-migration model for three species. *Syst. Biol.*, 66:379–398, 2017.

[46] J. Heled and A.J. Drummond. Bayesian inference of species trees from multilocus data. *Mol. Biol. Evol.*, 27: 570–580, 2010.

[47] Z. Yang and B. Rannala. Unguided species delimitation using DNA sequence data from multiple loci. *Mol. Biol. Evol.*, 31(12):3125–3135, 2014. doi: 10.1093/molbev/msu279.

[48] B. Rannala and Z. Yang. Efficient Bayesian species tree inference under the multispecies coalescent. *Syst. Biol.*, 66:823–842, 2017.

[49] T. Flouri, X. Jiao, B. Rannala, and Z. Yang. Species tree inference with BPP using genomic sequences and the multispecies coalescent. *Mol. Biol. Evol.*, 35(10): 2585–2593, 2018.

[50] J. Felsenstein. Evolutionary trees from DNA sequences: a maximum likelihood approach. *J. Mol. Evol.*, 17:368–376, 1981.

[51] J. Hey and R. Nielsen. Integration within the Felsenstein equation for improved Markov chain Monte Carlo methods in population genetics. *Proc. Natl. Acad. Sci. USA*, 104(8): 2785–2790, 2007.

[52] Z. Yang and B. Rannala. Bayesian species delimitation using multilocus sequence data. *Proc. Natl. Acad. Sci. USA*, 107:9264–9269, 2010.

[53] L. Liu and D.K. Pearl. Species trees from gene trees: reconstructing Bayesian posterior distributions of a species phylogeny using estimated gene tree distributions. *Syst. Biol.*, 56(3):504–514, 2007.

[54] F. Ronquist and J.P. Huelsenbeck. MrBayes 3: Bayesian phylogenetic inference under mixed models. *Bioinformatics*, 19:1572–1574, 2003.

[55] G. Jones. Algorithmic improvements to species delimitation and phylogeny estimation under the multispecies coalescent. *J. Math. Biol.*, 74:447–467, 2017.

[56] H.A. Ogilvie, R.R. Bouckaert, and A.J. Drummond. StarBEAST2 brings faster species tree inference and accurate estimates of substitution rates. *Mol. Biol. Evol.*, 34(8): 2101–2114, 2017.

[57] L. Liu and L. Yu. Estimating species trees from unrooted gene trees. *Syst. Biol.*, 60:661–667, 2011.

[58] J. Chifman and L.S. Kubatko. Quartet inference from SNP data under the coalescent model. *Bioinformatics*, 30:3317–3324, 2014.

[59] E. Sayyari and S. Mirarab. Fast coalescent-based computation of local branch support from quartet frequencies. *Mol. Biol. Evol.*, 33(7):1654–1668, 2016.

[60] H. Huang and L.L. Knowles. What is the danger of the anomaly zone for empirical phylogenetics? *Syst. Biol.*, 58: 527–536, 2009.

[61] L.N. Andersen, T. Mailund, and A. Hobolth. Efficient computation in the IM model. *J. Math. Biol.*, 68:1423–1451, 2014.

[62] T. Zhu and Z. Yang. Complexity of the simplest species tree problem. *Mol. Biol. Evol.*, 2021. doi: 10.1093/molbev/msab009.

[63] L. Liu, L. Yu, and D.K. Pearl. Maximum tree: a consistent estimator of the species tree. *J. Math. Biol.*, 60:95–106, 2010.

[64] A.D. Leaché and B. Rannala. The accuracy of species tree estimation under simulation: a comparison of methods. *Syst. Biol.*, 60(2):126–137, 2010.

[65] M. DeGiorgio and J.H. Degnan. Robustness to divergence time underestimation when inferring species trees from estimated gene trees. *Syst. Biol.*, 63:66–82, 2014.

[66] T.H. Jukes and C.R. Cantor. Evolution of protein molecules. In *Munro, H.N., ed. Mammalian Protein Metabolism*, pages 21–123. Academic Press, New York, 1969.

[67] S. Roch and M. Steel. Likelihood-based tree reconstruction on a concatenation of aligned sequence data sets can be statistically inconsistent. *Theor. Popul. Biol.*, 100:56–62, 2015.

[68] A. Kim and J.H. Degnan. PRANC: ML species tree estimation from the ranked gene trees under coalescence. *Bioinformatics*, 36(18):4819–4821, 2020. doi: 10.1093/bioinformatics/btaa605.

[69] E. J. Baack and L. H. Rieseberg. A genomic view of introgression and hybrid speciation. *Curr. Opin. Genet. Dev.*, 17(6):513–518, 2007. doi: 10.1016/j.gde.2007.09.001.

[70] J. Mallet, N. Besansky, and M.W. Hahn. How reticulated are species? *BioEssays*, 38(2):140–149, 2016.

[71] S.H. Martin and C.D. Jiggins. Interpreting the genomic landscape of introgression. *Curr. Opin. Genet. Dev.*, 47: 69–74, 2017.

[72] M.C. Fontaine, J.B. Pease, A. Steele, R.M. Waterhouse, D.E. Neafsey, I.V. Sharakhov, X. Jiang, A.B. Hall, F. Catteruccia, E. Kakani, S.N. Mitchell, Y.C. Wu, H.A. Smith, R.R. Love, M.K. Lawniczak, M.A. Slotman, S.J. Emrich, M.W. Hahn, and N.J. Besansky. Extensive introgression in a malaria vector species complex revealed by phylogenomics. *Science*, 347(6217):1258524, 2015.

[73] S.H. Martin, K.K. Dasmahapatra, N.J. Nadeau, C. Salazar, J.R. Walters, F. Simpson, M. Blaxter, A. Manica, J. Mallet, and C.D. Jiggins. Genome-wide evidence for speciation with gene flow in *Heliconius* butterflies. *Genome Res.*, 23

(11):1817–1828, 2013.
[74] R. Nielsen, J.M. Akey, M. Jakobsson, J.K. Pritchard, S. Tishkoff, and E. Willerslev. Tracing the peopling of the world through genomics. *Nature*, 541:302, 2017.
[75] A.D. Leaché, R.B. Harris, B. Rannala, and Z. Yang. The influence of gene flow on Bayesian species tree estimation: a simulation study. *Syst. Biol.*, 63(1):17–30, 2014.
[76] C. Solis-Lemus, M. Yang, and C. Ane. Inconsistency of species tree methods under gene flow. *Syst. Biol.*, 65(5): 843–851, 2016. doi: 10.1093/sysbio/syw030.
[77] C. Long and L.S. Kubatko. The effect of gene flow on coalescent-based species-tree inference. *Syst. Biol.*, 67: 770–785, 2018.
[78] X. Jiao, T. Flouri, B. Rannala, and Z. Yang. The impact of cross-species gene flow on species tree estimation. *Syst. Biol.*, 69:830–847, 2020. doi: 10.1093/sysbio/syaa001.
[79] R. Nielsen and J. Wakeley. Distinguishing migration from isolation: a Markov chain Monte Carlo approach. *Genetics*, 158:885–896, 2001.
[80] Y. Yu, J.H. Degnan, and L. Nakhleh. The probability of a gene tree topology within a phylogenetic network with applications to hybridization detection. *PLoS Genet.*, 8(4): e1002660, 2012. doi: 10.1371/journal.pgen.1002660.
[81] T. Flouri, X. Jiao, B. Rannala, and Z. Yang. A Bayesian implementation of the multispecies coalescent model with introgression for phylogenomic analysis. *Mol. Biol. Evol.*, 37(4):1211–1223, 2020.
[82] M. Notohara. The coalescent and the genealogical process in geographically structured populations. *J. Math. Biol.*, 29:59–75, 1990.
[83] H.B. Nath and R.C. Griffiths. The coalescent in two colonies with symmetric migration. *J. Math. Biol.*, 31: 841–852, 1993.
[84] H.M. Wilkinson-Herbots. Genealogy and subpopulation differentiation under various models of population structure. *J. Math. Biol.*, 37:535–585, 1998.
[85] P. Beerli and J. Felsenstein. Maximum-likelihood estimation of migration rates and effective population numbers in two populations using a coalescent approach. *Genetics*, 152:763–773, 1999.
[86] P. Beerli and J. Felsenstein. Maximum likelihood estimation of a migration matrix and effective population sizes in n subpopulations by using a coalescent approach. *Proc. Natl. Acad. Sci. U.S.A.*, 98:4563–4568, 2001.
[87] Y. Wang and J. Hey. Estimating divergence parameters with small samples from a large number of loci. *Genetics*, 184: 363–379, 2010.
[88] J. Hey. Isolation with migration models for more than two populations. *Mol. Biol. Evol.*, 27:905–920, 2010.
[89] J. Hey, Y. Chung, A. Sethuraman, J. Lachance, S. Tishkoff, V. C. Sousa, and Y. Wang. Phylogeny estimation by integration over isolation with migration models. *Mol. Biol. Evol.*, 35(11):2805–2818, 2018.
[90] I. Gronau, M.J. Hubisz, B. Gulko, C.G. Danko, and A. Siepel. Bayesian inference of ancient human demography from individual genome sequences. *Nature Genet.*, 43: 1031–1034, 2011.
[91] S. Zhu and J. Degnan. Displayed trees do not determine distinguishability under the network multispecies coalescent. *Syst. Biol.*, 66:283–298, 2017.
[92] J.H. Degnan. Modeling hybridization under the network multispecies coalescent. *Syst. Biol.*, 67(5):786–799, 2018.
[93] D. Wen and L. Nakhleh. Coestimating reticulate phylogenies and gene trees from multilocus sequence data. *Syst. Biol.*, 67(3):439–457, 2018.
[94] C. Zhang, H.A. Ogilvie, A.J. Drummond, and T. Stadler. Bayesian inference of species networks from multilocus sequence data. *Mol. Biol. Evol.*, 35:504–517, 2018.
[95] C. Solis-Lemus, P. Bastide, and C. Ane. PhyloNetworks: a package for phylogenetic networks. *Mol. Biol. Evol.*, 34 (12):3292–3298, 2017.
[96] Y. Yu, J. Dong, K.J. Liu, and L. Nakhleh. Maximum likelihood inference of reticulate evolutionary histories. *Proc. Natl. Acad. Sci. U.S.A.*, 111(46):16448–16453, 2014.
[97] G.R. Jones. Divergence estimation in the presence of incomplete lineage sorting and migration. *Syst. Biol.*, 68 (1):19–31, 2019.
[98] L.S. Kubatko. Identifying hybridization events in the presence of coalescence via model selection. *Syst. Biol.*, 58(5):478–488, 2009. doi: 10.1093/sysbio/syp055.
[99] C. Meng and L.S. Kubatko. Detecting hybrid speciation in the presence of incomplete lineage sorting using gene tree incongruence: a model. *Theor. Popul. Biol.*, 75:35–45, 2009.
[100] J. Zhu, Y. Yu, and L. Nakhleh. In the light of deep coalescence: revisiting trees within networks. *BMC Bioinform.*, 17:415, 2016.
[101] E.Y. Durand, N. Patterson, D. Reich, and M. Slatkin. Testing for ancient admixture between closely related populations. *Mol. Biol. Evol.*, 28:2239–2252, 2011.
[102] N. Patterson, P. Moorjani, Y. Luo, S. Mallick, N. Rohland, Y. Zhan, T. Genschoreck, T. Webster, and D. Reich. Ancient admixture in human history. *Genetics*, 192: 1065–1093, 2012.
[103] J.B. Pease and M.W. Hahn. Detection and polarization of introgression in a five-taxon phylogeny. *Syst. Biol.*, 64: 651–662, 2015.
[104] P.D. Blischak, J. Chifman, A.D. Wolfe, and L.S. Kubatko. HyDe: A python package for genome-scale hybridization detection. *Syst. Biol.*, 67(5):821–829, 2018.
[105] L.S. Kubatko and J. Chifman. An invariants-based method for efficient identification of hybrid species from large-scale genomic data. *BMC Evol. Biol.*, 19(1):112, 2019. doi: 10.1186/s12862-019-1439-7.
[106] C. Solis-Lemus and C. Ane. Inferring phylogenetic networks with maximum pseudolikelihood under incomplete lineage sorting. *PLoS Genet.*, 12(3):e1005896, 2016. doi: 10.1371/journal.pgen.1005896.
[107] S. Aeschbacher, J.P. Selby, J.H. Willis, and G. Coop. Population-genomic inference of the strength and timing of selection against gene flow. *Proc. Natl. Acad. Sci. U.S.A.*, 114:7061–7066, 2017.
[108] A.D. Kern and J. Hey. Exact calculation of the joint allele frequency spectrum for isolation with migration models. *Genetics*, 207:241–253, 2017.
[109] F. Pardi and C. Scornavacca. Reconstructible phylogenetic networks: Do not distinguish the indistinguishable. *PLoS Comput. Biol.*, 11(4):e1004135, 2015. doi: 10.1371/journal.pcbi.1004135.
[110] M. Stephens. Dealing with label switching in mixture models. *J. R. Statist. Soc. B.*, 62:795–809, 2000.
[111] J. Huang, T. Flouri, and Z. Yang. A simulation study to examine the information content in phylogenomic datasets under the multispecies coalescent model. *Mol. Biol. Evol.*, 37(11):3211–3224, 2020. doi: 10.1093/molbev/msaa166.
[112] M. Slatkin and J.L. Pollack. Subdivision in an ancestral species creates asymmetry in gene trees. *Mol. Biol. Evol.*, 25(10):2241–2246, 2008. doi: 10.1093/molbev/msn172.
[113] R.J. Costa and H. Wilkinson-Herbots. Inference of gene flow in the process of speciation: an efficient maximum-likelihood method for the isolation-with-initial-migration model. *Genetics*, 205(4):1597–1618, 2017. doi: 10.1534/genetics.116.188060.
